# Maternal satisfaction with breastfeeding: a systematic review with meta-analysis

**DOI:** 10.1590/0034-7167-2025-0028

**Published:** 2026-01-09

**Authors:** Ana Paula de Souza Martins Lemos, Wellington Francisco Rodrigues, Carlo José Freire de Oliveira, Divanice Contim, Jacqueline Faria de Oliveira, Mariana Torreglosa Ruiz

**Affiliations:** I Universidade Federal do Triângulo Mineiro. Uberaba, Minas Gerais, Brazil.; II Universidade Federal do Triângulo Mineiro, Hospital de Clínicas. Uberaba, Minas Gerais, Brazil.

**Keywords:** Personal Satisfaction, Breast Feeding, Weaning, Association, Systematic Review, Satisfacción Personal, Lactancia Materna, Destete, Asociación, Revisión Sistemática

## Abstract

**Objectives:**

to estimate the combined prevalence of maternal satisfaction with breastfeeding and associated factors.

**Methods:**

a systematic prevalence review, according to the PRISMA and JBI protocols. Searches were conducted in PubMed/MEDLINE, LILACS, Scopus, Embase, Web of Science, and CINAHL, using the “Breastfeeding” and “Personal Satisfaction” descriptors. Data were extracted, tabulated, and presented in a meta-synthesis, applying meta-analysis to quantitative data.

**Results:**

twelve studies published between 2003 and 2024 were included. The results indicated moderate maternal satisfaction with breastfeeding. Higher scores were observed in conditions favorable to breastfeeding and when breastfeeding was longer, and lower scores were observed in cases of early weaning, unmet expectations and intentions regarding breastfeeding, as well as complications in the process. Professional support contributed to satisfaction.

**Conclusions:**

factors that influence satisfaction with breastfeeding were revealed. However, the subjectivity of the concept and the multiplicity of measurement instruments may have contributed to the studies’ high heterogeneity.

## RESUMEN

**Objetivos:**

estimar la prevalencia combinada de la satisfacción materna con la lactancia materna y sus factores asociados.

**Métodos:**

se realizó una revisión sistemática de prevalencia según los protocolos PRISMA y JBI. Se realizaron búsquedas en las bases de datos PubMed/MEDLINE, LILACS, Scopus, Embase, *Web of Science* y CINAHL utilizando los descriptores “Lactancia Materna” y “Satisfacción Personal”. Los datos se extrajeron, tabularon y presentaron en una metasíntesis, aplicando un metaanálisis a los datos cuantitativos.

**Resultados:**

se incluyeron doce estudios publicados entre 2003 y 2024. Los resultados indicaron una satisfacción materna moderada con la lactancia materna. Se observaron puntuaciones más altas en condiciones favorables para la lactancia materna y cuando esta era más prolongada, y puntuaciones más bajas en casos de destete precoz, expectativas e intenciones incumplidas con respecto a la lactancia materna, así como complicaciones durante el proceso. El apoyo profesional contribuyó a la satisfacción.

**Conclusiones:**

se identificaron factores que influyen en la satisfacción con la lactancia materna. Sin embargo, la subjetividad del concepto y la multiplicidad de instrumentos de medición podrían haber contribuido a la alta heterogeneidad de los estudios.

## INTRODUCTION

Breastfeeding (BF) is recognized as a promoter and protector of child health and development^(1-3)^. BF is natural, nutritious, and sustainable^(4,5)^, capable of positively impacting maternal health in the long term as well as the family economy^(3-5)^.

BF contributes significantly to achieving the Sustainable Development Goals (SDGs) linked to good nutrition, food security, reduced inequalities for newborns, and gender equality and professional growth, when BF workers are supported to maintain it^(6)^.

The practice of exclusive breastfeeding (EBF) is sustainable, relating to the following SDGs: SDG 1 (“No hunger and poverty”), as it reduces family costs with formulas; SDG 2 (“Zero hunger”), as it has all the immune and nutritional components appropriate for the newborn; SDG 3 (“Good health and well-being”), as it contributes to maternal and child health in the short and long term; SDG 5 (“Gender equality”), when lactating women are supported to maintain BF and reconcile it with their work activities; SDG 12 (“Responsible consumption”), as it reduces the production of waste associated with artificial BF (cans, utensils); and SDG 13 (“Global climate”), as by reducing waste, the emission of gases that contribute to the greenhouse effect is reduced^(7,8)^. Thus, this study covers all the listed SDGs, as it aims to promote BF in its exclusive form.

However, BF is not an instinctive process, but rather a complex one permeated by various influences that reflect the decision to wean. Globally, 80% of newborns receive breast milk at some point in their lives, but 48% remain on EBF at six months, as recommended^(5)^. In Brazil, 96.2% of children were breastfed at least once in their lives, and 45.8% remain on EBF at six months of age^(9)^.

It is worth reflecting on maternal satisfaction with BF, since BF success has been assessed predominantly by its duration^(5,8)^, without considering the satisfaction of the woman involved. Thus, understanding the degree of satisfaction of lactating women and correlated factors helps to increase the effectiveness of BF as well as constituting an incentive for other women who live with nursing mothers satisfied with the lactation experience^(10)^.

In this regard, studies that seek to measure women’s degree of satisfaction with BF are still scarce^(11)^. This scarcity can be justified because women’s satisfaction with BF involves aspects often not observed by healthcare professionals, such as observation of BF, and to identify it, it needs to be questioned, involving bonding, active and non-judgmental listening, which requires advanced communication skills^(12)^.

Women with low levels of satisfaction with BF in the first month after birth are at greater risk of stopping BF before six months^(13)^. Therefore, when low maternal satisfaction with BF is detected, interventions should be implemented with women to support them and promote a pleasurable and lasting experience for the mother-baby dyad. Healthcare professionals should help mothers define, based on their perceptions, what constitutes successful BF, highlighting its strengths and weaknesses, and offering the necessary support to empower them to make the best decisions and achieve satisfaction with the process^(10)^. To this end, clear, accessible, welcoming, dialogic, and empathetic language should be used, taking care not to treat breast milk merely as a product, as this could negatively impact mothers’ feelings and their satisfaction with BF^(10)^.

Given the high rates of weaning among children, including early weaning, due to the benefits of BF for maternal and child health, society, and the achievement of the SDGs, and the influence of maternal satisfaction with BF on its maintenance, it is important to identify the prevalence of maternal satisfaction, which goes beyond BF duration and biological aspects, and can directly impact both the current and future BF experience. Furthermore, studies on BF satisfaction are recent and scarce, requiring further investigation.

## OBJECTIVES

To estimate the combined prevalence of maternal satisfaction with BF and associated factors.

## METHODS

### Ethical aspects

Since this study used publicly available data and did not involve human subjects, it did not require review by a Research Ethics Committee. However, it is important to note that the studies selected for the final sample were duly referenced.

### Design

This is a systematic prevalence review. According to the JBI, systematic reviews of prevalence or incidence data are becoming more important as public policymakers recognize the usefulness of synthesizing this type of information. They aim to inform and update social and healthcare professionals, public policymakers, and consumers for health decision-making, particularly regarding the current health burden and its projection for the future^(14)^.

The study was registered in the International Prospective Register of Systematic Reviews database, under registration CRD42024607191, structured according to the Preferred Reporting Items for Systematic reviews and Meta-Analyses (PRISMA) protocol^(15)^ and recommendations for systematic reviews of prevalence of JBI^(14)^.

### Study period and place

The review question was based on the Condition, Context, and Population (CoCoPop) strategy, establishing Co (Condition) for the prevalence of maternal satisfaction, Co (Context) for BF, and Pop (Population) for postpartum women. Based on these definitions, the review question was: What is the prevalence of maternal satisfaction with BF? Based on the analysis of the studies, the following questions were also asked: what are the associated factors? Do favorable conditions influence maternal satisfaction?

The searches were conducted independently on September 5 and 6, 2024, by two reviewers: a master’s student and a doctoral student. One reviewer has experience with search strategies, and both are specialists in maternal and child health. The search was validated by a librarian. Searches were conducted in US National Library of Medicine/National Institutes of Health (MEDLINE/PubMed), Web of Science (WoS), Excerpta Medica DataBASE (Embase), SciVerse Scopus, Cumulative Index to Nursing and Allied Health Literature (CINAHL), and Latin American and Caribbean Literature in Health Sciences (LILACS), correlating controlled descriptors from Medical Subject Headings (MeSH), CINAHL Headings, Embase Emtree, and Health Sciences Descriptors (DeCS): “Breastfeeding” and “Personal Satisfaction”. No date, language, or study design filters were applied. The search strategy development process followed the Peer Review of Electronic Search Strategies recommendations, and these strategies are presented in Chart 1 (Supplementary Material 1).

### Inclusion and exclusion criteria

Cross-sectional, longitudinal, cohort, or follow-up studies, without language or time frame limitations, that reported the prevalence of maternal satisfaction with BF or that provided data that allowed the calculation of such a measure, regardless of whether assessed as a primary outcome, and possible associated factors, were selected. Publications that did not meet the established eligibility criteria were excluded. The level of evidence was not considered an exclusion criterion, as this is a little-explored topic.

**Figure 1:**
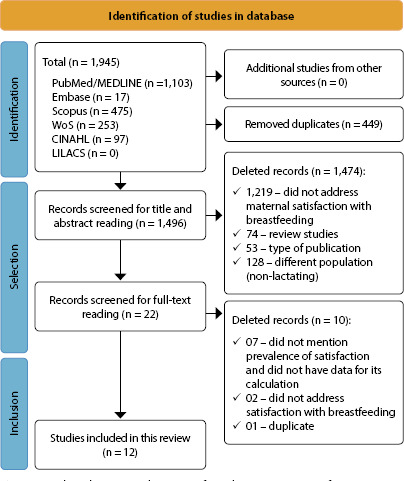
Flowchart according to Preferred Reporting Items for Systematic Reviews and Meta-Analysis

A total of 1,945 articles were identified in the six databases consulted. The PRISMA methodology was adopted^(15)^ and is presented in Figure 1. In the first stage, duplicates were removed (n = 449), and 1,474 articles were excluded after reading titles and abstracts because they did not address the study topic. The selected articles were then read in full. After reading, the final sample consisted of 12 studies.

### Study protocol

Study selection was performed independently by two researchers, and disagreements were resolved by consensus. There was no need to add a third reviewer at this stage. The selected articles were initially analyzed by reading the title and abstract, followed by full-text reading for final selection. The databases analyzed were selected in the order of PubMed, Embase, CINAHL, LILACS, WoS, and Scopus. The exclusion order followed the following criteria: duplicate articles; study design (reviews (secondary data); study design inappropriate to the question) (editorials); expert opinions; letters to the editor or article comments; case studies (single case report); guidelines, research protocols, and consensus statements; and those that did not answer the review question. Full texts were also selected in a paired and independent manner. Figure 1 illustrates the selection process for included studies.

### Analysis of results

Data were extracted as recommended by the JBI^(14)^, which included identification of the article, country, study setting or context, participant characteristics, groups, measured outcomes, and description of the main results, when cohort studies were included. For prevalence studies, article identification, country, year/timeframe for data collection, participant characteristics, measurement conditions and methods, and description of the main results were assessed. The extracted information was tabulated for data synthesis, and the analysis of results was descriptive, presenting a summary of each primary study included in this review.

The data were stored in Microsoft Excel® spreadsheets, and for the analyses, the R software version 4.4.2 was used, with the support of the “metafor” package to conduct meta-analyses and meta-regressions.

Meta-analyses were conducted with random-effects or mixed-effects models, using the between-study variance estimator (τ²) based on the Restricted Maximum Likelihood method. These models allowed integrating the results of included studies and addressing heterogeneity among them, estimating a combined prevalence with a 95% Confidence Interval (95% CI). Heterogeneity was assessed using the τ², I², and H² statistics. The significance of heterogeneity was tested with the Q statistic, allowing us to identify variations not explained by sampling variation.

Subgroup analyses were performed to explore the impact of different conditions (favorable or unfavorable to BF) on the prevalence of BF satisfaction. Subgroup analyses used mixed-effects models to estimate mean differences among groups, considering residual heterogeneity as a variable of interest.

The risk of publication bias was assessed using funnel plots and Egger’s test, which examines graph asymmetry. The funnel plot was used to visualize the relationship between the studies’ prevalence estimates and their standard errors. Graph symmetry indicates a low risk of publication bias, while asymmetries suggest potential bias problems. Egger’s test, in turn, statistically assessed the presence of asymmetry and was used as a visual and quantitative complement to the analysis.

Forest plots were generated for all analyses, illustrating the individual prevalence estimates from each study, the 95% CI, and the pooled estimate obtained by the random-effects model. These plots were adjusted to represent different conditions (specific favorable or unfavorable). For subgroup and meta-regression analyses, the plots were adapted to reflect the estimated differences between the groups and the predictive effects of assessed moderators. This approach combined robust quantitative synthesis techniques and methodological quality assessment tools, ensuring the reliability and transparency of the results presented.

All analyses were conducted respecting the statistical assumptions and considering the significant heterogeneity among included studies.

## RESULTS

### Studies characterization

Twelve studies were included in the analysis. The first publication dates from 2003^(16)^, and the last from 2024^(17)^, all in English. Three studies (25%)^(12,13,18)^ were produced in Brazil, and two (16.7%)^(19,20)^ in the United States. Australia^(16)^, Canada^(21)^, Scotland^(22)^, France^(23)^, Jordan^(17)^, Lebanon^(24)^ and Thailand^(25)^ were represented by one publication from each country (8.3%, respectively), showing a wide diversity of producing countries.

The cohort method was adopted in ten studies (83.3%)^(12,13,16-19,21-24)^. The cross-sectional design^(20,25)^ was used in two studies (16.7%). It is worth noting that one study^(25)^ is a cross-sectional study nested in a randomized clinical trial.

Most studies measured maternal satisfaction with BF using the Maternal Breastfeeding Evaluation Scale (MBFES), with versions translated and adapted for each country (eight studies – 66.7%)^(12,13,16-19,22,24)^. Three studies (25%)^(21,23,25)^ were based on Likert-type responses about satisfaction, and one study (8.3%)^(20)^ assessed satisfaction by the dichotomous response “yes” or “no”.

The application of JBI Tools for assessing methodological quality and risk of bias identified a low risk of bias in all included studies (with compliance with items and scores above 70%). However, despite good methodological quality, some items were not met in the studies, according to their design. This consideration applies to clinical trials, in which it was not possible to mask participants and researchers, since strategies/methods are visually different, which did not compromise the results or the quality of the studies analyzed.

In total, 5,182 dyads participated in the studies and were assessed for satisfaction with the BF process. Chart 2 presents the characteristics of the included studies.

**Chart 2:** Characteristics of cohort (n=10) and prevalence studies included in the review (n = 02), 2024

Characteristics of cohort studies included in the review (n=10)								
Study	Country	Setting/context	Participants	Group	Measured outcomes	Main results	Associated factors	JBI risk of bias
Bizon AMBL, Giugliani C & Giugliani ERJ, 2023^(13)^	Brazil	Cohort with dyads born in a public and a private maternity hospital in the city of Porto Alegre, RS, in 2016	213 dyads	Based on the median satisfaction score: women with scores below the median (<124) and above (>124)	Satisfaction with BF (measured by MBFES) in the first month and EBF rates	Women with satisfaction scores above the median had a mean EBF duration of 120 days (95% CI: 109-131), and women with satisfaction below the median had an EBF duration of 26 days (95% CI: 19-33) (p <0.001) Association between satisfaction and EBF discontinuation in the first month.	Negative association between satisfaction and interruption of BF in the first month.	11/11
Ahmed AH & Rojjanasrirat W, 2021^(19)^	United States	Dyads participating in the Women, Infants, and Children project, which assessed BF and its effects in the United States of America from 2014 to 2017	270 dyads	Dyads with late preterm birth (between 34 and 36 weeks and six days), early term birth (between 37 and 37 weeks and six days) and term birth (over 38 weeks) were compared.	Satisfaction with BF (measured by MBFES).	The mean satisfaction score was 116 (SD = 15.3) for the late preterm group, 114 (SD = 22.12) for the early term group, and 121.95 (SD = 16.86) for the full-term group. There were significant differences between the late preterm and full-term groups and between the early term and full-term groups, with an association between EBF and higher satisfaction scores at two weeks, two months, and five months.	Association with gestational age at birth and satisfaction with EBF.	11/11
Avilla *et al*., 2020^(12)^	Brazil	Cohort with dyads born in a public and a private maternity hospital in the city of Porto Alegre, RS	287 dyads	Based on average satisfaction scores: women with scores below average (<124) and above (>124)	Satisfaction with BF (measured by MBFES).	Satisfaction scores ranged from 63 to 145 (mean: 124).	Association between satisfaction with BF and postpartum depression. Satisfaction is 47% higher among women with negative screening for postpartum depression (adjusted PR: 1.47; 95% CI 1.01–2.16).	11/11
Nabulsi *et al*., 2021^(24)^	Lebanon	Birth cohort from a public, university, tertiary care hospital in Lebanon from 2018 to 2020	485 dyads	Type of BF in the third month: EBF (n = 172); mixed (n = 146) and artificial (n = 127)	Satisfaction with BF (measured by MBFES) and EBF.	Mean satisfaction score of 111.7 ± 13.6. The EBF rate during the study fell from 53.8% at two weeks to 38.4% at three months postpartum.	Positive correlations with maternal attitude towards BF (r = 0.30, p < 0.001), EBF at one (r = 0.27) and three months (r = 0.26, p < 0.001 for both), as well as with the longest previous EBF period (r = 0.27, p < 0.001).	11/11
Gregory *et al*., 2015^(20)^	United States	Survey administered by the Center for Disease Control between 2005 and 2007, with women recruited during prenatal and postpartum follow-up.	1,802 dyads	Gestational expectations regarding BF met (n = 627) or not (n = 1,175)	Satisfaction with BF measured by “yes” or “no” responses and fulfillment of prenatal expectations (“yes” or “no”).	65.2% of women had their expectations not met, but 26.3% were satisfied with EBF duration; 34.8% had their expectations met; and 79.1% were satisfied with EBF duration. EBF duration was longer in satisfied women (7.2 vs. 3.6 months; t 931 = 16.49; p < 0.001).	Younger women, multiparous women, those who self-identified as Black or Hispanic, or who had completed high school had higher rates of satisfaction with duration. Women with postpartum depression and obesity had lower rates of satisfaction. Satisfaction was associated with meeting expectations (OR: 10.56; 95% CI: 7.67-14.55).	11/11
Cooke *et al*., 2003^(16)^	Australia	Cohort of women who gave birth in three Sydney public hospitals in 1999	241 dyads	Intercurrences in the BF process	Satisfaction with BF (measured by MBFES).	Complications during BF significantly decreased the mean satisfaction score (maternal satisfaction: 52.8 ± 1.6 vs. 56.5 ± 1.6, p<0.01).	Belief of insufficient milk (p < 0.01); sore nipples (p = 0.01); engorgement (p = 0.02) and other complications (p < 0.01). Lower satisfaction scores were associated with weaning, and the perception of insufficient milk was a predictor of weaning between two and six weeks.	11/11
Laberére *et al*., 2012^(23)^	France	Cohort of births occurring in eight French maternity hospitals in 2012 (public and private)	907 dyads	Women who indicated that they were very satisfied with BF (n = 822) and dissatisfied (n = 85)	Satisfaction with BF (measured on a Likert scale – very dissatisfied/dissatisfied; very satisfied/satisfied).	637 (70%) were very satisfied; 185 (21%) were satisfied; 58 (6%) were dissatisfied; and 27 (3%) were very dissatisfied.	Dissatisfaction: duration shorter than expected; smokers; those who offered nipples since birth; and those who had complications during the BF process.	11/11
El-Shaer *et al*., 2024^(17)^	Jordan	Cohort of births occurring in an institution part of the BFHI from 2021 to 2023	160 dyads	Comparison of indicators before and after implementation of the BFHI	Satisfaction with BF (measured by MBFES).	Satisfaction scores ranged from 26 to 124, with a mean of 93.59 ± 15.83.	Higher satisfaction scores with BF were observed after the implementation of the BFHI.	11/11
Symon *et al*., 2012^(22)^	Scotland	Birth cohort from an eastern Scottish town	292 dyads	Scores compared according to BF intention	Satisfaction with BF (measured by questionnaire by Leff *et al*., 1994, MBFES).	The average satisfaction score was 50.3 ± 5.10, ranging from 18 to 59 points.	Higher satisfaction scores were observed among women who had prenatal intentions to breastfeed their child for more than eight weeks.	11/11
Lamontagne *et al*., 2009^(21)^	Canada	Longitudinal study of Quebec lactating women	86 dyads	Women who sought BF support services due to complications (n = 52) and women who did not have access to the service (n = 34)	Satisfaction with BF (measured using a Likert-type scale), with responses ranging from 1 to 5.	In the group that received support, 59.6% were very satisfied; 19.2% were satisfied; and 21.2% were dissatisfied or very dissatisfied. In the group that did not receive support, 44.1% were satisfied; 17.7% were satisfied; and 38.2% were dissatisfied or very dissatisfied.	Women who received professional support had greater chances of satisfaction with BF (OR = 4.17; 95% CI = 1.31-13.22).	11/11
**Characteristics of prevalence studies included in the review (n = 02)**								
**Study**	**Country**	**Data collection year**	**Participants**	**Focus/method**	**Main results**	**Associated factors**	**JBI risk of bias**	
De Senna *et al*., 2020^(18)^	Brazil	2016	287 dyads	Satisfaction with BF (measured by MBFES)/cross-sectional study nested within the cohort	Satisfaction scores in the first month postpartum ranged from 63 to 145, with a median of 124 and a mean score of 120 ± 14.	Women with brown (pardo) and black skin (PR 1.33; 95% CI 1.05; 1.69) and those who lived with a partner (PR 1.75; 95% CI 1.05; 2.94), with higher scores, women who planned to breastfeed for 12 months or more were more satisfied (PR 1.48, 95% CI 1.02;2.17) and women who did not report low milk supply or cracked nipples had greater satisfaction (PR 1.47; 95% CI 1.03; 2.10; and PR 1.29, 95% CI 1.01; 1.65, respectively).	9/9	
Puapornpong *et al*.^(25)^	Thailand	2017	152 post-cesarean dyads who were instructed to breastfeed laterally (n = 76) or maintain a dorsal position – laid back (n = 76)	Satisfaction measured using a Likert scale from 1 to 5/randomized clinical trial	Satisfaction scores for women BF sideways were higher (4.0 ± 0.5) than scores for the laid-back group (3.8 ± 0.7) for comfort, ease of positioning, and use of the position for a long period of time.	Positioning and comfort during BF contributed to satisfaction.	12/13 (due to the nature of the intervention, it was not possible to blind participants)	

95% CI - 95% Confidence Interval; BF - breastfeeding; BFHI - Baby-Friendly Hospital Initiative; PR - Prevalence Ratio; MBFES - Maternal Breastfeeding Evaluation Scale.

### Factors associated with maternal satisfaction with breastfeeding

The included studies measured a wide variety of variables associated with maternal satisfaction with BF, with considerable heterogeneity in objectives and measurement instruments.

Women with satisfaction scores below the median were more likely to discontinue BF within the first month after birth^(13)^. Similar results were found when measuring EBF indicators at 1 and 3 months of age^(24)^. Both publications point to an association between BF satisfaction and duration, including its exclusive form.

Regarding sociodemographic variables, higher satisfaction scores were observed in younger women^(20)^, multiparous women^(20)^, women who self-identified as black^(18,20)^ or Hispanic^(20)^, women who lived with a partner^(18)^ and women who had completed at least high school^(20)^.

Concerning clinical variables associated with BF satisfaction, a study showed an association of lower scores in smokers^(23)^ and obese women^(20)^. It was also observed that women with negative screening for postpartum depression had higher satisfaction scores, indicating the influence of depressive symptoms on low satisfaction^(12,20)^.

Regarding variables associated with birth, higher satisfaction scores were observed in women who reached full-term gestational age when compared to late preterm or premature pregnancies^(19)^. A study also found that women who were cared for in an institution accredited as a Baby-Friendly Hospital had higher satisfaction scores with BF after implementing the strategy^(17)^.

Variables associated with BF were predictors of higher or lower satisfaction scores. Women who had freedom of positioning and felt comfortable BF had higher satisfaction scores^(25)^. Those who had been offered nipples since birth had lower scores, indicating dissatisfaction^(23)^.

Breast complications during BF were predictors of lower satisfaction scores^(16,18,23)^. The belief of insufficient milk production, sore nipples, and breast engorgement, among other complications, were associated with lower satisfaction scores^(16)^. It is noteworthy that lower satisfaction scores were associated with early weaning, reinforced the perception of insufficient production, and were predictors of weaning between two and six weeks postpartum^(16)^. However, a study found that, when faced with complications, receiving professional support and overcoming difficulties was associated with higher maternal satisfaction scores^(21)^. Studies point to the influence of complications and professional support to overcome them and the impact on maternal satisfaction.

Finally, it is worth noting that satisfaction was associated with meeting expectations regarding BF^(18,20,23)^ as well as with the intention to breastfeed^(22)^. Figure 2 (Supplementary Material 3) summarizes the factors associated with the highest and lowest maternal satisfaction scores with BF.

### Prevalence of satisfaction with breastfeeding

**Figure 3:**
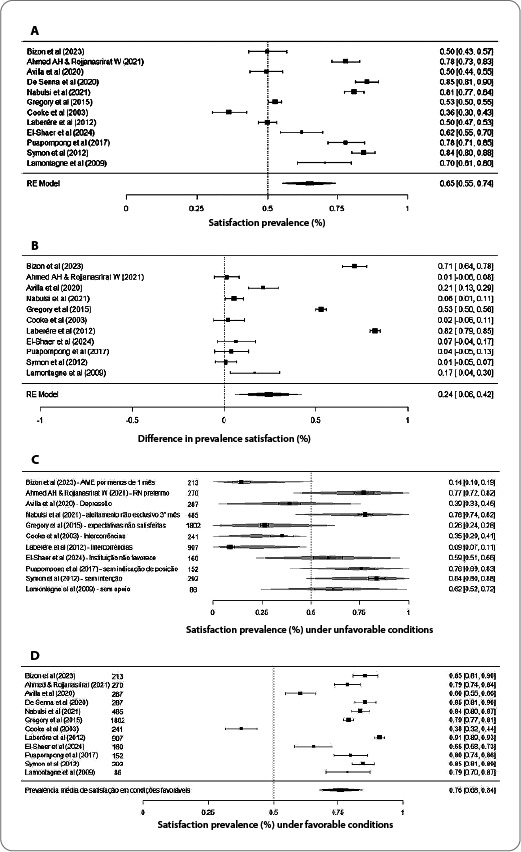
A. Meta-analysis comparing satisfaction (%) prevalence estimates for each study included in the meta-analysis. B. Meta-analysis comparing the differences in satisfaction prevalence with breastfeeding between favorable and unfavorable conditions. C. Meta-analysis comparing satisfaction prevalence with breastfeeding under different unfavorable conditions. D. Meta-analysis comparing satisfaction prevalence with breastfeeding under favorable conditions for each study included in the meta-analysis.

The mean prevalence of maternal satisfaction with BF was 64.83% (CI = 55.32–74.34%), but high heterogeneity was observed between studies (I2 = 98%). The CI analysis indicates a consistently positive prevalence of satisfaction across studies, with highly robust statistical significance (p < 0.001). These results suggest an overall prevalence of moderate satisfaction among participants in the included studies, although the high heterogeneity indicates substantial variations in the reported prevalence across individual studies. The graphical representation is presented in Figure 3 (3A).

### Differences in satisfaction under favorable and unfavorable conditions for breastfeeding

The prevalence of maternal satisfaction was compared under favorable and unfavorable conditions for BF. A significant mean difference in satisfaction prevalence of 24.27 percentage points was observed in favor of favorable BF conditions (CI = 6.39 - 42.15; p = 0.0078). However, high heterogeneity was again observed among the studies (I² = 99%). The representation of the differences is shown in Figure 3 (3B).

### Satisfaction with breastfeeding under unfavorable conditions

Subgroup analysis was conducted to assess satisfaction prevalence with BF under unfavorable conditions. The mean prevalence was 78.10% (CI = 0.43 - 1.14), again with high heterogeneity among the analyzed studies (I² = 98%). Maternal satisfaction was relatively high, although negatively moderated by some specific conditions, such as EBF for less than one month (-0.64; CI = -1.14 - -0.12), unmet expectations and intentions (-0.52; CI = -1.02 - -0.01), and complications during the BF process (-0.56; CI = -1.00 - -0.12).

Other conditions, such as indicators of postpartum depression, care in an institution not accredited by the Baby-Friendly Hospital Initiative, birth of premature newborns, lack of professional support during the process, freedom to position the newborn at the mother’s breast, and no intention to breastfeed, presented estimates that were not statistically significant (p > 0.05). The graphical representation of the influence of unfavorable conditions on maternal satisfaction is shown in Figure 3 (3C).

### Satisfaction and favorable conditions for breastfeeding

Similarly, satisfaction prevalence under conditions favorable to BF was compared. The estimated mean prevalence of satisfaction was 75.99% (CI = 67.6 - 84.3%). However, high heterogeneity was observed (I² = 98%). The results indicate high satisfaction under favorable conditions with statistical significance (p <0.001). The comparison of the prevalence of maternal satisfaction with BF under favorable conditions is shown in Figure 3 (3D).

### Publication bias

**Figure 4:**
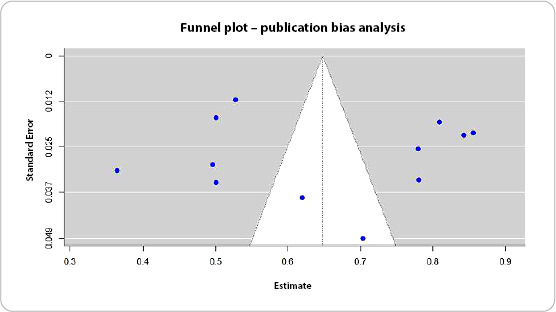
Funnel plot representing publication bias analysis in the meta-analysis of prevalence of satisfaction with breastfeeding

The presence of publication bias was assessed using a funnel plot and Egger’s test (Figure 4). The funnel plot showed a symmetrical distribution of studies around the pooled estimate, indicating a low risk of publication bias. Egger’s test was not significant (p = 0.80), supporting the absence of asymmetry in the funnel plot. These findings suggest that the meta-analysis results are robust and not influenced by publication bias, reinforcing the reliability of the pooled prevalence estimates.

## DISCUSSION

The results indicated a moderate prevalence of maternal satisfaction with BF, but with substantial variations depending on specific and individual circumstances. Higher satisfaction scores were observed in conditions favorable to BF and when BF lasted longer. Early weaning, unmet expectations and intentions regarding BF, and complications during the process were associated with lower satisfaction scores. Furthermore, professional support helped overcome difficulties, contributing to better scores.

A validity study of the Spanish version of MBFES with 690 lactating women showed that the instrument is valid and reliable for assessing maternal satisfaction with BF, which was positively associated with longer EBF duration, as in the present study, but negatively influenced by the perceived difficulty in maintaining BF while working^(26)^.

Professional support and assistance are essential for maintaining BF, especially EBF, in challenging situations, such as premature newborns. Continuity and a support network after discharge are essential for overcoming difficulties and increasing BF duration^(27)^, which can contribute to a positive BF experience.

Intercurrences during the BF process can act as barriers to BF initiation or continuation. Breast problems such as lesions, engorgement, mastitis, difficulties with positioning and latching, and the belief of low or insufficient milk production are the most common complications. In this context, professional support is essential to resolve and overcome these complications^(28)^. When complications are not overcome, women tend to be less satisfied with the BF experience.

Maternal intention to breastfeed, which corresponds to predisposition to breastfeed, as well as expectations, when met, were associated with BF satisfaction. BF intention is an individual, complex, and progressive process that will shape a woman’s behavior and begins during pregnancy, directly influenced by a series of factors^(29)^. Similarly to the results of the present study, intention and satisfaction are positively influenced by previous experience with BF^(30,31)^, living with a partner and receiving support from them^(30)^, not having a smoking habit^(30)^, and higher educational level^(30)^.

Lower satisfaction scores were associated with mixed and/or artificial BF, stressful situations, and premature births. Higher satisfaction scores were associated with longer BF duration and professional support^(32)^.

When BF support is offered to women, it increases BF duration and exclusivity. Characteristics of effective support include being provided by trained professionals, preferably during prenatal or postnatal care while still in the Rooming-In facility, including scheduled and ongoing home or healthcare service visits, and ensuring women are informed that they can access the available support network when needed, tailored to the environment and needs of women and their family. Furthermore, in-person support is more likely to be successful with women who practice EBF^(33)^.

Thus, the study leads us to reflect that satisfaction is directly associated with BF duration, the intentions and expectations met, as well as professional support, which is essential for overcoming unfavorable conditions for its practice.

We emphasize that satisfaction was measured with different instruments. The most common, the widely used MBFES, has different scores and scales depending on the country where the cross-cultural adaptation was conducted, which may contribute to the studies’ heterogeneity. The original instrument proposed by Leff *et al*. has 30 items and its score ranges from 30 to 150^(34)^. Two American studies^(19,20)^ adopted this same score, indicating that the version was adapted to the country’s context. A Scottish study adopted a scale of 12 to 60 points, using the same scale translated and validated for the country^(22)^. Even when faced with different scores, it is important to highlight that translation and cross-cultural adaptation of an instrument must consider whether the original version is valid and useful for measuring the intended outcome, and in these cases, it must be translated and adapted according to the language, but respecting the sociocultural context of the country^(35)^, which justifies the wide variation in the sum and items of the instrument, contributing to the studies’ heterogeneity.

Finally, it is worth reflecting on the subjectivity of the concept of satisfaction and the multiplicity of factors that can influence it. The romanticization of BF and choices and decisions regarding it can lead to significant maternal guilt^(36)^, and without support, it can increase feelings of dissatisfaction, loneliness, and responsibility for the experience. It is also worth mentioning that the World Health Organization, in its guidelines for a positive postnatal experience, emphasizes professional support and assistance, especially in cases of complications during the BF process, and the use of BF counseling, emphasizing active listening to BF women^(37)^.

### Study limitations

Limitations include the subjectivity of the concept of satisfaction and the multiplicity of instruments used to measure it, which may have contributed to the high heterogeneity of studies. Furthermore, recent concern with the topic is evident, which may compromise the comparability of results. However, while these limitations also provide an opportunity for further studies on the topic.

### Contributions to nursing and health

Given the evidence presented, the importance of support from a trained team becomes clear, especially in unfavorable conditions, so that BF women can overcome barriers and difficulties and feel satisfied with their experience. The need for more homogeneous studies with controlled variables regarding methodology is emphasized, preferably randomized controlled clinical trials that explore the factors associated with satisfaction, contributing to BF protection and promotion as well as to a positive maternal experience.

## CONCLUSIONS

A moderate prevalence of satisfaction with BF was observed, but with substantial variations depending on the specificities. Favorable conditions for BF contribute to higher satisfaction scores with the experience. However, early weaning, unmet expectations and intentions, and complications during the BF process contribute to lower satisfaction scores. However, it is important to note the high heterogeneity of studies and methods of measuring satisfaction.

## Data Availability

The research data are available within the article.
