# Supplementary material for: Maternal satisfaction with breastfeeding: a systematic review with meta-analysis
Source: Rev Bras Enferm. 2026 Jan 9;78(Suppl 3):e20250028. doi: 10.1590/0034-7167-2025-0028 (PMC12798862; doi:10.1590/0034-7167-2025-0028)
Supplement: Translation

# Translation: Satisfação materna com o aleitamento materno: revisão sistemática com metanálise

**DOI:** 10.1590/0034-7167-2025-0028pt

**Authors:** Ana Paula de Souza Martins Lemos, Wellington Francisco Rodrigues, Carlo José Freire de Oliveira, Divanice Contim, Jacqueline Faria de Oliveira, Mariana Torreglosa Ruiz

**Affiliations:** I Universidade Federal do Triângulo Mineiro. Uberaba, Minas Gerais, Brazil.; II Universidade Federal do Triângulo Mineiro, Hospital de Clínicas. Uberaba, Minas Gerais, Brazil.

**Keywords:** Satisfação Pessoal, Aleitamento Materno, Desmame, Associação, Revisão Sistemática

## Abstract

**Objetivos:**

estimar a prevalência combinada de satisfação materna com o aleitamento materno e fatores associados.

**Métodos:**

revisão sistemática de prevalência, segundo protocolo PRISMA e JBI. Realizaram-se buscas nas bases PubMed/MEDLINE, LILACS, Scopus, Embase, *Web of Science* e CINAHL, usando os descritores “Aleitamento Materno” e “Satisfação Pessoal”. Dados foram extraídos, tabulados e apresentados em metassíntese, aplicando-se metanálise para dados quantitativos.

**Resultados:**

incluíram-se 12 estudos publicados entre 2003 e 2024. Resultados apontaram moderada satisfação materna em relação à amamentação. Observaram-se maiores escores em condições favoráveis à amamentação e quando a amamentação foi mais prolongada, e menores escores em casos de desmame precoce, expectativas e intenções não atendidas em relação à amamentação, bem como complicações no processo. O apoio/suporte profissional contribuiu para satisfação.

**Conclusões:**

desvelaram-se fatores que influenciam a satisfação com a amamentação. Contudo, a subjetividade do conceito e a multiplicidade de instrumentos para mensurar podem ter contribuído para alta heterogeneidade dos estudos.

## INTRODUÇÃO

O aleitamento materno (AM) é reconhecidamente promotor e protetor da saúde e do desenvolvimento infantil^(1-3)^. O AM é natural, nutritivo e sustentável^(4,5)^, capaz de impactar positivamente a saúde materna a longo prazo, assim como a economia familiar^(3-5)^.

A amamentação contribui significantemente para alcance dos Objetivos do Desenvolvimento Sustentável (ODS) vinculados à boa nutrição, à segurança alimentar, à redução das desigualdades para os neonatos, e à igualdade de gênero e crescimento profissional, quando lactantes trabalhadoras são apoiadas para sua manutenção^(6)^.

A prática do aleitamento exclusivo (AME) é sustentável, relacionando-se ao seguintes ODS: ODS 1 (“Erradicação da fome e pobreza”), pois reduz custos familiares com fórmulas; ODS 2 (“Fome zero”), pois possui todos os componentes imunes e nutricionais adequados ao neonato; ODS 3 (“Saúde e bem-estar”), pois contribui para a saúde materna e infantil a curto e longo prazo; ODS 5 (“Igualdade de gênero”), quando as lactantes são apoiadas a manter a amamentação e conciliar com suas atividades laborais; ODS 12 (“Consumo responsável”), pois reduz a produção de descartes associados ao aleitamento artificial (latas, utensílios); e ODS 13 (“Clima mundial”), pois, ao reduzir os descartes, reduz-se a emissão de gazes que contribuem para o efeito estufa^(7,8)^. Desta forma, este estudo contempla todos os ODS elencados, pois visa à promoção do AM em sua forma exclusiva.

Contudo, amamentar não é um processo instintivo, mas sim complexo e permeado por diversas influências que refletem na decisão pelo desmame. Mundialmente, 80% dos recém-nascidos recebem o leite materno em algum momento da vida, mas 48% permanecem em AME ao sexto mês, conforme a recomendação^(5)^. No Brasil, 96,2% das crianças foram amamentadas ao menos uma vez na vida e 45,8% permanecem em AME ao sexto mês de vida^(9)^.

Cabe a reflexão no que concerne à satisfação materna com a amamentação, uma vez que o êxito da amamentação tem sido avaliado predominantemente pela sua duração^(5,8)^, sem considerar a satisfação da mulher envolvida. Desse modo, compreender o grau de satisfação da lactante e os fatores correlacionados ajuda a aumentar a eficácia do AM, bem como constitui um estímulo para outras mulheres que convivem com nutrizes satisfeitas com a experiência da lactação^(10)^.

Nesse sentido, estudos que buscam mensurar o grau de satisfação das mulheres em amamentar ainda são escassos^(11)^. Essa escassez pode ser justificada porque a satisfação das mulheres em amamentar envolve aspectos muitas vezes não observados pelos profissionais de saúde, como a observação da mamada, sendo que, para identificá-la, necessita ser questionada, envolvendo vínculo, escuta ativa e sem julgamento, o que requer habilidades avançadas de comunicação^(12)^.

Mulheres com baixos níveis de satisfação com a amamentação no primeiro mês após o parto apresentam maior risco de interrompê-la antes de seis meses^(13)^. Assim, quando detectado baixo nível de satisfação materna em amamentar, intervenções devem ser realizadas com as mulheres a fim de acolhê-las e promover uma experiência prazerosa e duradoura para a díade mãe-bebê. Profissionais de saúde devem ajudar as mães a definirem, a partir da percepção delas, o que seria uma amamentação bem-sucedida, destacando seus acertos e pontos positivos, e oferecendo o apoio necessário, de forma a emponderá-la, para que tome as melhores decisões e resulte em satisfação com o processo^(10)^. Para isso, deve ser utilizada linguagem clara, acessível, acolhedora, dialógica e empática, tendo o cuidado de não tratar o leite materno apenas como um produto, uma vez que isso poderia impactar negativamente os sentimentos da mãe e sua satisfação com a amamentação^(10)^.

Tendo em vista os altos índices de desmame nas crianças, incluindo sua ocorrência precoce, devido aos benefícios do AM para a saúde materno-infantil, para a sociedade e para o alcance dos ODS, e à influência da satisfação materna com o AM para a sua manutenção, faz-se relevante identificar a prevalência de satisfação materna, que vai além da duração e dos aspectos biológicos do aleitamento, podendo impactar diretamente tanto a experiência atual quanto a futura com a amamentação. Além disso, os estudos sobre satisfação com a amamentação são recentes e escassos, demandando maiores investigações.

## OBJETIVOS

Estimar a prevalência combinada de satisfação materna com o AM e fatores associados.

## MÉTODOS

### Aspectos éticos

Por se tratar de estudo que utilizou dados de domínio público e não envolveu seres humanos, não houve necessidade de apreciação pelo Comitê de Ética em Pesquisa. Porém, cabe ressaltar que os estudos selecionados para a amostra final foram devidamente referenciados.

### Desenho

Trata-se de revisão sistemática de prevalência. De acordo com o JBI, revisões sistemáticas de dados de prevalência ou incidência estão se tornando mais importantes à medida que os formuladores de políticas públicas percebem a utilidade de sínteses desse tipo de informação. Têm como objetivo informar e atualizar profissionais da área social e da saúde, formuladores de políticas públicas e consumidores, para a tomada de decisão em saúde, particularmente no que diz respeito à carga de saúde no momento presente e sua projeção para o futuro^(14)^.

O estudo foi registrado na base de dados *International Prospective Register of Systematic Reviews*, sob o registro CRD42024607191, estruturado de acordo com o protocolo *Preferred Reporting Items for Systematic reviews and Meta-Analyses* (PRISMA)^(15)^ e recomendações para revisões sistemáticas de prevalência do JBI^(14)^.

### Período e local do estudo

A questão de revisão se baseou na estratégia Condição, Contexto e População (CoCoPop), estabelecendo Co (Condição) para prevalência de satisfação materna, Co (Contexo), para amamentação, e Pop (População), para puérperas. Com base nessas definições, a questão de revisão foi: qual a prevalência de satisfação materna com a amamentação? A partir da análise dos estudos, também se questionou: quais os fatores associados? As condições favoráveis influenciam a satisfação materna?

As buscas foram realizadas em 05 e 06 de setembro de 2024, de forma independente, por dois revisores: um mestrando e um doutor. Um revisor tem experiência com estratégia de busca, e ambos são especialistas na área da saúde materno-infantil. A busca foi validada por um bibliotecário. Foram realizadas buscas nas bases de dados *US National Library of Medicine National Institutes of Health* (MEDLINE/PubMed), *Web of Science* (WoS), *Excerpta Medica DataBASE* (Embase), SciVerse Scopus, *Cumulative Index to Nursing and Allied Health Literature* (CINAHL) e Literatura Latino-Americana e do Caribe em Ciências da Saúde (LILACS), correlacionando descritores controlados do *Medical Subject Headings* (MeSH), do CINAHL *Headings*, do Embase *Emtree* e do Descritores em Ciências da Saúde (DeCS): “Aleitamento Materno” e “Satisfação Pessoal”. Não foram aplicados filtros de data, idioma e/ou desenho de estudo. O processo de elaboração das estratégias de busca atendeu às recomendações do *Peer Review of Electronic Search Strategies* e essas estratégias são apresentadas no Quadro 1 (Material Suplementar 1).

### Critérios de inclusão e de exclusão

Foram selecionados estudos transversais, longitudinais, coortes ou estudos de seguimento (*follow-up*), sem limitação de idioma e recorte de tempo, que apresentaram a prevalência de satisfação materna com o AM ou que ofertaram dados que permitissem o cálculo de tal medida, independentemente se avaliada como desfecho primário, e possíveis fatores associados. Foram excluídas publicações que não atendessem aos critérios de elegibilidade estabelecidos. O nível de evidência não foi considerado critério de exclusão, por se tratar de temática pouco explorada.

Identificaram-se 1.945 artigos nas seis bases de dados consultadas. A metodologia PRISMA foi adotada^(15)^ e está apresentada na Figura 1. Na primeira etapa, as duplicatas foram removidas (n = 449) e 1.474 artigos foram excluídos após a leitura dos títulos e resumos, por não retratarem o tema de estudo. Após, os artigos selecionados foram lidos na íntegra. Após a leitura, a amostra final foi composta por 12 estudos.

**Figura 1:** Fluxograma segundo *Preferred Reporting Items for Systematic Reviews and Meta-Analysis*

### Protocolo do estudo

A seleção dos estudos foi realizada de modo independente por dois pesquisadores, e as discordâncias foram resolvidas por consenso. Não houve necessidade de acréscimo do terceiro revisor nesta etapa. A análise dos artigos selecionados foi realizada, em uma primeira etapa, com a leitura do título e resumo, seguida de leitura na íntegra para a seleção final dos artigos. A ordem das bases de dados analisadas foi PubMed, Embase, CINAHL, LILACS, WoS e Scopus. A ordem de exclusões seguiu os seguintes critérios: artigos duplicados; tipo de estudo – revisões (dados secundários); desenho de estudo inadequado à questão - editoriais; opiniões de especialistas; cartas ao editor ou comentários de artigos; estudos de caso (único caso reportado); *guidelines*, protocolos de pesquisa e consensos; e os que não respondiam à questão de revisão. Os textos completos também foram selecionados de modo pareado e independente. A Figura 1 ilustra o processo de seleção dos estudos incluídos.

### Análise dos resultados

Os dados foram extraídos conforme recomendação do JBI^(14)^, que incluíram a identificação do artigo, país, cenário ou contexto do estudo, características dos participantes, grupos, desfechos medidos e descrição dos principais resultados, quando foram incluídos estudos de coorte. Para os estudos de prevalência, foram avaliados a identificação do artigo, o país, o ano/prazo para coleta de dados, as características dos participantes, as condições e métodos de medição, e a descrição dos principais resultados. As informações extraídas foram tabuladas para síntese dos dados, e a análise dos resultados foi descritiva, apresentando uma síntese de cada estudo primário incluído nesta revisão.

Os dados foram armazenados em planilhas do *Microsoft Excel* ^®^, e para as análises, foi utilizado o *software R,* versão 4.4.2, com o suporte do pacote “metafor” para condução das meta-análises e meta-regressões.

As meta-análises foram conduzidas com modelos de efeitos aleatórios ou mistos, utilizando o estimador de variância entre os estudos (τ²) baseado no método *Restricted Maximum Likelihood*. Esses modelos permitiram integrar os resultados dos estudos incluídos e abordar a heterogeneidade entre eles, estimando uma prevalência combinada com Intervalo de Confiança de 95% (IC95%). A heterogeneidade foi avaliada por meio das estatísticas τ², I² e H². A significância da heterogeneidade foi testada com a estatística Q, permitindo identificar variações não explicadas pela variação amostral.

Foram realizadas análises de subgrupo para explorar o impacto de diferentes condições (favoráveis ou desfavoráveis ao aleitamento) sobre a prevalência de satisfação com o aleitamento. As análises de subgrupo utilizaram modelos de efeitos mistos para estimar as diferenças médias entre os grupos, considerando a heterogeneidade residual como uma variável de interesse.

A avaliação do risco de viés de publicação foi realizada por meio de gráficos de funil e do teste de Egger, que examina a assimetria no gráfico. O gráfico de funil foi utilizado para visualizar a relação entre as estimativas de prevalência dos estudos e seus erros padrão. A simetria do gráfico indica baixo risco de viés de publicação, enquanto assimetrias sugerem possíveis problemas de viés. O teste de Egger, por sua vez, avaliou estatisticamente a presença de assimetria, sendo utilizado como complemento visual e quantitativo para a análise.

Os *forests plots,* ou gráficos de floresta, foram gerados para todas as análises, ilustrando as estimativas individuais de prevalência de cada estudo, os IC95% e a estimativa combinada obtida pelo modelo de efeitos aleatórios. Esses gráficos foram ajustados para representar diferentes condições (favoráveis ou desfavoráveis específicas). Para as análises de subgrupo e meta-regressão, os gráficos foram adaptados para refletir as diferenças estimadas entre os grupos e os efeitos preditivos dos moderadores avaliados. Essa abordagem combinou técnicas robustas de síntese quantitativa e ferramentas de avaliação da qualidade metodológica, garantindo a confiabilidade e a transparência dos resultados apresentados.

Todas as análises foram conduzidas respeitando os pressupostos estatísticos e considerando a heterogeneidade significativa entre os estudos incluídos.

## RESULTADOS

### Caracterização dos estudos

Foram incluídos na análise 12 estudos. A primeira publicação data de 2003^(16)^, e a última, de 2024^(17)^, todas no idioma inglês. Três estudos (25%)^(12,13,18)^ foram produzidos no Brasil, e dois (16,7%)^(19,20)^, nos Estados Unidos. Austrália^(16)^, Canadá^(21)^, Escócia^(22)^, França^(23)^, Jordânia^(17)^, Líbano^(24)^ e Tailândia^(25)^ foram representados por uma publicação de cada país (8,3%, respectivamente), mostrando ampla diversidade de países produtores.

O método de coorte foi adotado em dez estudos (83,3%)^(12,13,16-19,21-24)^. Já o recorte transversal^(20,25)^ foi utilizado em dois estudos (16,7%). Vale ressaltar que um estudo^(25)^ é um estudo transversal aninhado em um ensaio clínico randomizado.

A maioria dos estudos mensurou a satisfação materna com a amamentação através do *Maternal Breastfeeding Evaluation Scale* (MBFES), com suas versões traduzidas e adaptadas para cada país (oito estudos – 66,7%)^(12,13,16-19,22,24)^. Três estudos (25%)^(21,23,25)^ se basearam em respostas do tipo Likert sobre a satisfação, e um estudo (8,3%)^(20)^ avaliou a satisfação pela resposta dicotômica “sim” ou “não”.

A aplicação de ferramentas para avaliação da qualidade metodológica e do risco de viés do JBI *Tools* possibilitou identificar baixo risco de viés em todos os estudos inclusos (com atendimento dos itens e escores acima de 70%). Contudo, mesmo com boa qualidade metodológica, alguns dos itens não foram atendidos nos estudos, de acordo com seu desenho. Esta consideração é aplicada aos ensaios clínicos, nos quais não foi possível mascarar participantes e pesquisadores, uma vez que as estratégias/métodos são visualmente diferentes, o que não comprometeu os resultados ou a qualidade dos estudos analisados.

Ao todo, participaram dos estudos 5.182 díades, avaliadas quanto à satisfação com o processo de amamentação. As características dos estudos inclusos estão apresentadas no Quadro 2.

**Quadro 2:** Características dos estudos de coorte (n=10) e de prevalência incluídos na revisão (n = 02), 2024

Características dos estudos de coorte inclusos na revisão (n=10)								
Estudo	País	Cenário/contexto	Participantes	Grupo	Desfechos mensurados	Resultados principais	Fatores associados	Risco de viés do JBI
Bizon AMBL, Giugliani C & Giugliani ERJ, 2023^(13)^	Brasil	Coorte com díades nascidas em uma maternidade pública e uma privada no município de Porto Alegre, RS, no ano de 2016	213 díades	Baseado na mediana do escore de satisfação: mulheres com escores abaixo da mediana (<124) e acima (>124)	Satisfação com a amamentação (mensurada pelo MBFES) no primeiro mês e índices de AME	Mulheres com escores de satisfação acima da mediana tiveram duração média de AME de 120 dias (IC95%: 109-131), e mulheres com satisfação abaixo da mediana tiveram duração de AME de 26 dias (IC95%: 19 -33) (p <0,001) Associação entre a satisfação e interrupção do AME no primeiro mês.	Associação negativa entre satisfação e interrupção do aleitamento no primeiro mês.	11/11
Ahmed AH & Rojjanasrirat W, 2021^(19)^	Estados Unidos	Díades participantes do projeto *Women, Infants, and Children*, que avalia AM e seus efeitos nos Estados Unidos da América, de 2014 a 2017	270 díades	Comparadas díades com nascimento pré-termo tardio (entre 34 e 36 semanas e seis dias), termo precoce (entre 37 e 37 semanas e seis dias) e termo (acima de 38 semanas)	Satisfação com a amamentação (mensurada pelo MBFES).	O escore médio de satisfação foi de 116 (DP = 15,3) para o grupo pré-termo tardio, 114 (DP = 22,12), para o grupo termo precoce, e 121,95 (DP = 16,86), para o grupo termo. Houve diferenças significantes entre o grupo pré-termo tardio e o grupo termo e entre o termo precoce e o grupo termo, com associação entre AME e maiores escores de satisfação entre duas semanas, dois meses e cinco meses.	Associação com a idade gestacional ao nascimento e satisfação com o AME.	11/11
Avilla *et al*., 2020^(12)^	Brasil	Coorte com díades nascidas em uma maternidade pública e uma privada no município de Porto Alegre, RS	287 díades	Baseado na média dos escores de satisfação: mulheres com escores abaixo da média (<124) e acima (>124)	Satisfação com a amamentação (mensurada pelo MBFES).	Os escores de satisfação variaram de 63 a 145 (média: 124).	Associação entre satisfação com AM e depressão pós-parto. A satisfação é 47% maior entre mulheres com *screening* negativo para depressão pós-parto (RP ajustada: 1,47; 95%CI 1,01–2,16).	11/11
Nabulsi *et al*., 2021^(24)^	Líbano	Coorte de nascimentos de um hospital público, universitário e de assistência terciária no Líbano, de 2018 a 2020	485 díades	Tipo de AM no terceiro mês: AME (n = 172); misto (n = 146) e artificial (n = 127)	Satisfação com a amamentação (mensurada pelo MBFES) e exclusividade do AM.	Pontuação média de satisfação de 111,7 ±13,6. A taxa de AME durante o estudo caiu de 53,8%, em duas semanas, para 38,4%, três meses após o parto.	Correlações positivas com a atitude materna em relação à amamentação (r = 0,30, p < 0,001), AME em um (r = 0,27) e aos três meses (r = 0,26, p < 0,001 para ambos), bem como com o período de AME anterior mais longo (r = 0,27, p < 0,001).	11/11
Gregory *et al*., 2015^(20)^	Estados Unidos	*Survey* aplicado pelo Centro de Controle de Doenças, entre 2005 e 2007, com mulheres recrutadas durante o pré-natal e seguimento pós-parto	1.802 díades	Expectativas gestacionais em relação à amamentação atendidas (n = 627) ou não (n = 1.175)	Satisfação com o AM medida por resposta tipo “sim” ou “não” e atendimento de expectativa pré-natal (“sim” ou “não”).	65,2% das mulheres tiveram sua expectativa não atendida, contudo 26,3% ficaram satisfeitas com a duração do AME; 34,8% tiveram a expectativa atendida; e 79,1% ficaram satisfeitas com a duração do AME. A duração do AME foi maior em mulheres satisfeitas (7,2 x 3,6 meses; t 931 = 16,49; p < 0,001).	Mulheres mais jovens, multíparas, que se declaravam raça negra ou hispânica, ou tinham concluído o ensino médio tinham maiores índices de satisfação com a duração. Mulheres com depressão pós-parto e obesas tinham menores índices de satisfação. A satisfação foi associada ao cumprimento das expectativas (OR: 10,56; IC95%: 7,67 14,55).	11/11
Cooke *et al*., 2003^(16)^	Austrália	Coorte de mulheres que tiveram partos em três hospitais públicos de Sidney em 1999	241 díades	Intercorrências no processo de amamentação	Satisfação com a amamentação (mensurada pelo MBFES).	Intercorrências durante a amamentação diminuíram significativamente a pontuação média de satisfação (satisfação materna: 52,8 ± 1,6 x 56,5 ± 1,6, p< 0,01).	Crença de leite insuficiente (p < 0,01); mamilos doloridos (p = 0,01); ingurgitamento (p = 0,02) e outras intercorrências (p < 0,01). Menores escores de satisfação se associaram ao desmame, e a percepção do leite insuficiente foi preditora do desmame entre duas e seis semanas.	11/11
Laberére *et al*., 2012^(23)^	França	Coorte de nascimentos ocorridos em oito maternidades francesas no ano de 2012 (públicas e privadas)	907 díades	Mulheres que indicaram estar muito satisfeitas com a amamentação (n = 822) e insatisfeitas (n = 85)	Satisfação com a amamentação (mensurada por escala tipo Likert – muito insatisfeitas/insatisfeitas; muito satisfeitas/satisfeitas).	637 (70%) estavam muito satisfeitas; 185 (21%) estavam satisfeitas; 58 (6%) estavam insatisfeitas; e 27 (3%) estavam muito insatisfeitas.	Insatisfação: duração inferior à expectativa; tabagistas; que ofertaram bicos desde o nascimento; e que tiveram intercorrências no processo de amamentação.	11/11
El-Shaer *et al*., 2024^(17)^	Jordânia	Coorte de nascimentos ocorridos em uma instituição parte da IHAC de 2021 a 2023	160 díades	Comparação dos indicadores antes e após a implementação da IHAC	Satisfação com a amamentação (mensurada pelo MBFES).	Os escores de satisfação variaram entre 26 e 124, com média de 93,59 ± 15,83.	Observaram-se maiores escores de satisfação com o AM após a implementação da IHAC.	11/11
Symon *et al*., 2012^(22)^	Escócia	Coorte de nascimentos de uma cidade do leste da Escócia	292 díades	Comparados escores segundo a intenção em amamentar	Satisfação com a amamentação (mensurada por questionário de Leff *et al*., 1994, MBFES).	O escore médio de satisfação foi de 50,3± 5,10, variando de 18 a 59 pontos.	Observaram-se maiores escores de satisfação entre mulheres que tinham intenção pré-natal de amamentar seu filho por mais de oito semanas.	11/11
Lamontagne *et al*., 2009^(21)^	Canadá	Estudo longitudinal com lactantes de Quebec	86 díades	Mulheres que buscaram serviço de apoio ao AM por intercorrências (n = 52) e mulheres que não tiveram acesso ao serviço (n = 34)	Satisfação com a amamentação (mensurada por meio de escala do tipo Likert), com respostas variando de 1 a 5.	No grupo que recebeu apoio, 59,6% estavam muito satisfeitas; 19,2% estavam satisfeitas; e 21,2% estavam insatisfeitas ou muito insatisfeitas. No grupo que não recebeu apoio, 44,1% estavam satisfeitas; 17,7% estavam satisfeitas; e 38,2% estavam insatisfeitas ou muito insatisfeitas.	Mulheres que receberam apoio profissional tiveram maiores chances de satisfação com a amamentação (OR = 4,17; IC95% = 1,31-13,22).	11/11
Características dos estudos de prevalência incluídos na revisão (n = 02)								
Estudo	País	Ano da coleta dos dados	Participantes	Foco/método	Resultados principais	Fatores associados	Risco de viés do JBI	
De Senna *et al*., 2020^(18)^	Brasil	2016	287 díades	Satisfação com a amamentação (mensurada pelo MBFES)/estudo transversal aninhado à coorte	Os escores de satisfação no primeiro mês pós-parto variaram de 63 a 145, com mediana de 124 e uma pontuação média de 120 ± 14.	Mulheres com pele parda (parda) e preta (RP 1,33; IC95% 1,05; 1,69) e aquelas que viviam com companheiro (RP 1,75; IC95% 1,05; 2,94), com maiores escores, mulheres que planejavam amamentar por 12 meses ou mais ficaram mais satisfeitas (RP 1,48, IC95% 1,02;2,17) e mulheres que não relataram baixa oferta de leite ou rachaduras nos mamilos apresentaram maior satisfação (RP 1,47; IC95% 1,03; 2,10; e RP 1,29, IC95% 1,01; 1,65, respectivamente).	9/9	
Puapornpong *et al*.^(25)^	Tailândia	2017	152 díades pós-cesárea que foram orientadas a amamentar lateralmente (n = 76) ou manter posição dorsal – *laid back* (n = 76)	Satisfação mensurada a partir de escala tipo Likert de 1 a 5/ensaio clínico randomizado	Os índices de satisfação das mulheres que amamentavam lateralmente foram maiores (4,0 ± 0,5) do que as pontuações do grupo *laid back* (3,8± 0,7) em conforto, facilidade de posicionamento e uso da posição por um longo período de tempo.	O posicionamento e o conforto na amamentação contribuíram para a satisfação.	12/13 (devido à natureza da intervenção, não foi possível cegar participantes)	

IC95% - Intervalo de Confiança de 95%; AM – aleitamento materno; IHAC - Iniciativa Hospital Amigo da Criança; RP – Razão de Prevalência; MBFES - Maternal Breastfeeding Evaluation Scale.

### Fatores associados à satisfação materna em amamentar

Os estudos inclusos mensuraram grande diversidade de variáveis associadas à satisfação materna em amamentar, com grande heterogeneidade de objetivos e instrumentos de mensuração.

Mulheres com escores de satisfação abaixo da mediana tinham maiores chances de interromper a amamentação ainda no primeiro mês após o nascimento^(13)^. Resultados semelhantes foram encontrados ao mensurar indicadores de AME com 1 e 3 meses de idade da criança^(24)^. Ambas as publicações apontam para a associação entre a satisfação e a duração do AM, incluindo sua forma exclusiva.

Em relação às variáveis sociodemográficas, observaram-se maiores escores de satisfação em mulheres mais jovens^(20)^, multíparas^(20)^, que se autodeclaravam negras^(18,20)^ ou hispânicas^(20)^, que viviam com companheiro^(18)^ e que tinham concluído ao menos o ensino médio^(20)^.

Quanto às variáveis clínicas associadas à satisfação com o aleitamento, estudo apontou associação de menores escores em mulheres tabagistas^(23)^ e entre mulheres obesas^(20)^. Observou-se, adicionalmente, que mulheres com *screening* negativo para depressão pós-parto possuíam maiores escores de satisfação, indicando influência dos sintomas depressivos na baixa satisfação^(12,20)^.

Em relação às variáveis associadas ao nascimento, observaram-se maiores escores de satisfação em mulheres que atingiram o termo da idade gestacional quando comparadas a gestações pré-termo tardias ou prematuras^(19)^. Estudo também apontou que mulheres que foram assistidas em instituição credenciada como Hospital Amigo da Criança apresentaram maiores escores de satisfação com a amamentação após a implementação da estratégia^(17)^.

Já as variáveis associadas à amamentação foram preditoras para maiores ou menores escores de satisfação. Mulheres com liberdade de posicionamento e que se sentiam confortáveis ao amamentar apresentaram maiores escores de satisfação^(25)^. Já as que ofertavam bicos desde o nascimento possuíam menores escores, indicando insatisfação^(23)^.

As intercorrências mamárias durante a amamentação foram preditoras para menores escores de satisfação^(16,18,23)^. A crença de produção láctea insuficiente, os mamilos doloridos e o ingurgitamento mamário, entre outras intercorrências, foram associados a menores escores de satisfação^(16)^. Ressalta-se que os menores escores de satisfação foram associados ao desmame precoce, reforçaram a percepção de produção insuficiente e foram preditores para o desmame entre duas e seis semanas pós-parto^(16)^. No entanto, estudo apontou que, diante de intercorrências, receber apoio profissional e superar as dificuldades foi associado a maiores escores de satisfação materna^(21)^. Os estudos apontam a influência das intercorrências e do apoio profissional para superá-las e o impacto na satisfação materna.

Por fim destaca-se que a satisfação se associou ao atendimento das expectativas em relação à amamentação^(18,20,23)^, assim como com a intenção em amamentar^(22)^. A Figura 2 (Material Suplementar 3) sintetiza os fatores associados aos maiores e menores escores de satisfação materna com o AM.

### Prevalência de satisfação com o aleitamento materno

A prevalência média de satisfação materna com o AM foi de 64,83% (IC = 55,32 – 74,34%), mas observa-se elevada heterogeneidade entre os estudos (I^2^ = 98%). A análise do IC indica uma prevalência de satisfação consistentemente positiva entre os estudos, com significância estatística altamente robusta (p < 0,001). Esses resultados sugerem uma prevalência geral de satisfação moderada entre as participantes dos estudos incluídos, embora a alta heterogeneidade indique variações substanciais nas prevalências relatadas entre os estudos individuais. A representação gráfica está apresentada na Figura 3 (3A).

**Figura 3:** A. Metanálise comparando as estimativas de prevalência de satisfação (%) para cada estudo incluído na meta-análise. B. Metanálise comparando as diferenças na prevalência de satisfação com o aleitamento entre condições favoráveis e não favoráveis. C. Metanálise comparando prevalência de satisfação com o aleitamento sob diferentes condições desfavoráveis. D. Metanálise comparando prevalência de satisfação com o aleitamento em condições favoráveis para cada estudo incluído na meta-análise.

### Diferenças de satisfação em condições favoráveis e desfavoráveis ao aleitamento materno

Comparou-se a prevalência de satisfação materna em condições favoráveis e desfavoráveis com o AM. Observou-se uma diferença média de prevalência significativa de satisfação de 24,27 pontos percentuais a favor das condições favoráveis ao aleitamento (IC = 6,39 - 42,15; p = 0,0078). No entanto, novamente observou-se alta heterogeneidade entre os estudos (I² = 99%). A representação das diferenças está representada na Figura 3 (3B).

### Satisfação com o aleitamento materno em condições desfavoráveis

A análise de subgrupo foi conduzida para avaliar a prevalência de satisfação com o AM em condições desfavoráveis. A prevalência média foi de 78,10% (IC = 0,43 - 1,14), novamente com alta heterogeneidade entre os estudos analisados (I² = 98%). A satisfação materna foi relativamente alta, embora moderada negativamente por algumas condições específicas, como AME por período inferior a um mês (-0,64; IC = -1,14 - -0,12), expectativas e intenções não satisfeitas (-0,52; IC = -1,02 - -0,01), e intercorrências durante o processo de AM (-0,56; IC = -1,00 - -0,12).

Outras condições, como indicativos de depressão pós-parto, assistência em instituição não credenciada à Iniciativa Hospital Amigo da Criança, nascimento de neonato prematuros, ausência de apoio e suporte profissional no processo, liberdade para posicionamento do recém-nascido no seio materno e não intenção em amamentar ao seio, apresentaram estimativas que não foram estatisticamente significativas (p > 0,05). A representação gráfica da influência de condições desfavoráveis na satisfação materna está posta na Figura 3 (3C).

### Satisfação e condições favoráveis ao aleitamento materno

Da mesma forma, realizou-se a comparação da prevalência de satisfação em condições favoráveis ao AM. A prevalência média estimada de satisfação foi de 75,99% (IC= 67,6 -84,3%). Contudo, observou-se alta heterogeneidade (I² = 98%). Os resultados apontam para satisfação elevada em condições favoráveis com significância estatística (p <0,001). A comparação da prevalência de satisfação materna com AM em condições favoráveis está representada na Figura 3 (3D).

### Viés de publicação

A presença de viés de publicação foi avaliada utilizando o gráfico de funil e o teste de Egger (Figura 4). O gráfico de funil apresentou uma distribuição simétrica dos estudos em torno da estimativa combinada, indicando baixo risco de viés de publicação. O teste de Egger não foi significativo (p = 0,80), corroborando a ausência de assimetria no gráfico de funil. Esses achados sugerem que os resultados da meta-análise são robustos e não sofreram influência de viés de publicação, reforçando a confiabilidade das estimativas combinadas de prevalência.

**Figura 4:** Gráfico de funil representando a análise de viés de publicação na meta-análise de prevalência de satisfação com o aleitamento

## DISCUSSÃO

Os resultados apontaram prevalência moderada de satisfação materna em relação à amamentação, porém com substanciais variações a depender de condições específicas e individuais. Observaram-se maiores escores de satisfação em condições favoráveis ao AM e quando a duração é maior. O desmame precoce, as expectativas e intenções não atendidas em relação ao AM, como as intercorrências no processo, foram associados a menores escores de satisfação. Além disso, observa-se que o apoio e suporte profissional auxiliaram na superação das dificuldades, contribuindo para melhores escores.

Estudo de validação da versão espanhola da MBFES com 690 lactantes apontou que o instrumento é válido e confiável para avaliar a satisfação materna com o AM, que foi associada positivamente à maior duração do AME, tal como no presente estudo, mas influenciada negativamente pela dificuldade percebida em manter amamentação concomitante com o trabalho^(26)^.

O apoio e suporte profissional são essenciais para manutenção do AM, principalmente na forma exclusiva, em situações desafiadoras, nos casos de neonatos prematuros. A continuidade e rede de apoio após a alta são primordiais para superar as dificuldades e aumentar a duração do AM^(27)^, que pode contribuir para uma experiência positiva associada à amamentação.

Já as intercorrências no processo de amamentação podem se impor como barreiras para o início ou continuidade do AM. Problemas mamários como lesões, ingurgitamento, mastite, dificuldades no posicionamento e com a pega do neonato, e a crença de baixa ou insuficiente produção láctea constituem as intercorrências mais comuns. Nesse contexto, o suporte e apoio profissional são fundamentais para sanar e superar as intercorrências^(28)^. Diante de intercorrências não superadas, as mulheres tendem a ter menor satisfação com a experiência de amamentar.

A intenção materna em amamentar, que corresponde à predisposição em amamentar, assim como a expectativa, quando atendidas, foram associadas à satisfação com o aleitamento. A intenção em amamentar é um processo individual, complexo e progressivo de construção que moldará o comportamento da mulher e que tem seu início durante a gestação, influenciada diretamente por uma série de fatores^(29)^. Da mesma forma que os resultados do presente estudo, a intenção e a satisfação são influenciadas positivamente pela experiência prévia com a amamentação^(30,31)^, viver com companheiro e receber apoio do mesmo^(30)^, não ter hábito de fumar^(30)^, e nível educacional superior^(30)^.

Menores escores de satisfação foram associados ao aleitamento misto e/ou aleitamento artificial, a situações estressantes, e a nascimentos prematuros. Já os maiores escores de satisfação se associaram à maior duração do AM e ao suporte e apoio profissional recebido^(32)^.

Quando o apoio à amamentação é oferecido às mulheres, aumentam-se a duração e a exclusividade da amamentação. As características de um apoio eficaz incluem que seja oferecido por profissionais capacitados, preferencialmente durante os cuidados pré-natais ou pós-natais ainda durante a internação no Alojamento Conjunto, que inclua visitas domiciliares ou nos serviços de saúde agendadas e contínuas, e que as mulheres sejam esclarecidas de que podem acessar a rede de apoio disponível quando necessário, e que seja adaptado ao ambiente e às necessidades da mulher e sua família. Além disso, o apoio presencial possui maior probabilidade de sucesso com as mulheres que praticam AME^(33)^.

Assim, o estudo nos leva a refletir que a satisfação está diretamente associada à duração da amamentação, às intenções e expectativas atendidas, assim como ao apoio/suporte profissional, essencial para a superação de condições desfavoráveis à sua prática.

Destacamos que a satisfação foi mensurada com diferentes instrumentos. O mais frequente, o MBFES, amplamente utilizado, possui pontuações e escalas diferentes de acordo com o país em que foi realizada a adaptação transcultural, o que pode contribuir para a heterogeneidade dos estudos. O instrumento original proposto por Leff *et al*. possui 30 itens e sua pontuação varia de 30 a 150^(34)^. Dois estudos americanos^(19,20)^ adotaram essa mesma pontuação, indicando que a versão foi adaptada ao contexto do país. Já estudo escocês adotou escala de 12 a 60 pontos, utilizando-se a mesma escala traduzida e validada para o país^(22)^. Mesmo diante de escores diferentes, é importante destacar que a tradução e adaptação transcultural de um instrumento devem considerar se a versão original é válida e útil para mensurar o desfecho pretendido, e nesses casos, deve ser traduzido e adaptado de acordo com o idioma, mas respeitando-se o contexto sociocultural do país^(35)^, o que justifica a ampla variação na somatória e nos itens do instrumento, contribuindo para a heterogeneidade dos estudos.

Por fim, cabe a reflexão a respeito da subjetividade do conceito de satisfação e da multiplicidade de fatores que podem influenciá-la. A romantização da amamentação e escolhas e decisões em relação à mesma podem levar à grande culpabilização materna^(36)^, e sem apoio, pode aumentar a sensação de insatisfação, solidão e responsabilização com a experiência. Cita-se ainda que a Organização Mundial da Saúde, em suas diretrizes para experiência pós-natal positiva, enfatiza o suporte e apoio profissional, principalmente em casos de intercorrências no processo de aleitamento, e o uso do aconselhamento em AM, enfatizando a escuta ativa da mulher que amamenta^(37)^.

### Limitações do estudo

Como limitação, podemos citar a subjetividade do conceito satisfação e a multiplicidade de instrumentos para mensurá-la, o que pode ter contribuído para a alta heterogeneidade dos estudos. Ademais, nota-se preocupação recente com a temática, o que pode comprometer a comparabilidade dos resultados. Porém, ao mesmo tempo que se constituem em limitantes, tornam-se uma oportunidade de desenvolvimento de novos estudos sobre a temática.

### Contribuições para as áreas da enfermagem e saúde

Diante das evidências apresentadas, fica evidente a importância do apoio e suporte de uma equipe capacitada, principalmente em condições desfavoráveis, para que a lactante possa transpor barreiras e dificuldades e se sinta satisfeita com a própria experiência. Salienta-se a necessidade de realização de estudos mais homogêneos e com variáveis controladas quanto à metodologia, preferencialmente investigações com ensaios clínicos randômicos e controlados, que explorem os fatores associados à satisfação, contribuindo para a proteção e promoção do AM, assim como para a experiência materna positiva.

## CONCLUSÕES

Observou-se prevalência de moderada satisfação com o AM, porém com variações substanciais, de acordo com as especificidades. Condições favoráveis para o AM contribuem para maiores escores de satisfação com a experiência. Já condições de desmame precoce, expectativas e intenções não satisfeitas, e intercorrências no processo de amamentação contribuem para menores escores de satisfação. Contudo, atenta-se para a alta heterogeneidade dos estudos e formas de mensuração da satisfação.

## Data Availability

Os dados da pesquisa estão disponíveis no corpo do artigo.
